# CANFOR Portuguese version: validation study

**DOI:** 10.1186/1471-244X-13-157

**Published:** 2013-05-30

**Authors:** Miguel Talina, Stuart Thomas, Ana Cardoso, Pedro Aguiar, Jose M Caldas de Almeida, Miguel Xavier

**Affiliations:** 1 CEDOC, Faculdade de Ciências Médicas, Universidade Nova de Lisboa, Campo Mártires da Pátria, Lisbon 1169-056, Portugal; 2 School of Psychology & Psychiatry, Monash University, and Victorian Institute of Forensic Mental Health, Melbourne, VIC, Australia; 3 Escola Nacional de Saúde Pública, Universidade Nova de Lisboa, Lisbon, Portugal

**Keywords:** Forensic psychiatry, Needs assessment, Mental disorders, Mentally disordered offenders, Prisoners, Prison

## Abstract

**Background:**

The increase in prisoner population is a troublesome reality in several regions of the world. Along with this growth there is increasing evidence that prisoners have a higher proportion of mental illnesses and suicide than the general population. In order to implement strategies that address criminal recidivism and the health and social status of prisoners, particularly in mental disordered offenders, it is necessary to assess their care needs in a comprehensive, but individual perspective. This assessment must include potential harmful areas like comorbid personality disorder, substance misuse and offending behaviours. The Camberwell Assessment of Need – Forensic Version (CANFOR) has proved to be a reliable tool designed to accomplish such aims. The present study aimed to validate the CANFOR Portuguese version.

**Methods:**

The translation, adaptation to the Portuguese context, back-translation and revision followed the usual procedures. The sample comprised all detainees receiving psychiatric care in four forensic facilities, over a one year period. A total of 143 subjects, and respective case manager, were selected. The forensic facilities were chosen by convenience: one prison hospital psychiatric ward (n=68; 47.6%), one male (n=24; 16.8%) and one female (n=22; 15.4%) psychiatric clinic and one civil security ward (n=29; 20.3%), all located nearby Lisbon. Basic descriptive statistics and Kappa weighted coefficients were calculated for the inter-rater and the test-retest reliability studies. The convergent validity was evaluated using the Global Assessment of Functioning and the Brief Psychiatric Rating Scale scores.

**Results:**

The majority of the participants were male and single, with short school attendance, and accused of a crime involving violence against persons. The most frequent diagnosis was major depression (56.1%) and almost half presented positive suicide risk. The reliability study showed average Kappa weighted coefficients of 0.884 and 0.445 for inter-rater and test-retest agreement, respectively. The convergent validity study presented highly significant correlations between unmet needs scores, GAF and BPRS scores.

**Conclusions:**

The CANFOR Portuguese version revealed similar psychometric properties to the original English version. Moreover, the results of the reliability and validity studies indicate that the tool is appropriate for individual care needs assessment and as a guide for the mental health and social interventions in forensic psychiatric services.

## Background

The growth of the prison population along with the increasing number of prisoners with mental disorders is one of the great concerns of the political and health authorities [1]. Review of 63 mental morbidity prevalence studies shows that one out of every seven prisoners (14.3%) in the western countries has a psychotic disorder or major depression [2]. In Europe, a study on mental disturbances in prisons of thirteen countries revealed that about five per cent of the prisoners met criteria for a psychotic disorder and about one in four presented an affective or anxiety disorder. When including those with drug abuse, 63% of them met criteria for a mental disorder [3]. These rates are significantly higher than those found in the community. According to Reed [4], the reasons for this high prevalence include higher risk of arrest for people with mental disorders alleged to have offended, inadequate coverage by court assessment schemes, too few psychiatric beds, and poor identification of mental disorders during the prison reception process. On the other hand, there are many factors in prisons that can have deleterious effects on mental health, including: overcrowding, various forms of violence, enforced solitude, or, conversely, lack of privacy, lack of meaningful activity, isolation from social networks, insecurity about future prospects (work, relationships, etc.), and inadequate health services in prisons, especially mental health services. The increased risk of suicide in prisons - often related to depression - is, unfortunately, one common manifestation of the cumulative effects of these factors [5]. Therefore, it is not surprising that mental health care needs are very high and represent a significant on-going challenge for service providers [2,3]. Nowadays there is broad consensus of the importance of addressing the needs of care of the mentally disordered offenders (MDOs), who, like the whole prison population, have higher proportions of medical and social problems compared to the community population and are subject to some form of social exclusion [2,6-9]. Whilst the social and clinical needs of MDOs are in many ways similar to those of general psychiatric patients, there are also differences that must be assessed. In particular, more emphasis must be placed on certain areas such as co-morbid personality disorder, substance misuse and offending behaviours [10,11].

There are several instruments developed to measure individual needs in general psychiatric populations. The Camberwell Assessment of Need (CAN) [12] is one of the most frequently used and potentially valuable tools for this purpose [13]. In order to acknowledge the differences between civil and forensic psychiatric populations, Thomas and colleagues developed a version based on the structure of CAN that was targeted to the forensic mental health service users: the Camberwell Assessment of Need – forensic version (CANFOR) [14]. The CANFOR maintain the same 22 domains of CAN plus three new domains related to treatment acquiescence and offending behaviour: “Treatment”, “Sexual Offences” and “Arson”. There are three versions of the published tool (the Research, Clinical and Short Versions) as well as a training manual [15].

### The situation in Portuguese prisons

The Portuguese prison system is administered by the General-Directorate of Prison Services, an organization that depends on the Ministry of Justice. There are 56 prison facilities throughout the territory, with near 12,000 places. As in many other countries, the number of prisoners has increased steadily over the last decades. Since 1974 (prison population: 2,519) the number of prisoners has increased more than 4.5 times [16]. At the beginning of the 70s, Portugal had one of the highest rates of prisoners in Western Europe: 132.8 per 100,000 inhabitants [17,18]. However, in recent years, the rate of prison population per 100,000 inhabitants has decreased, standing at 104.4 in 2009 [19]. This ratio places Portugal at the medium ranking in the European context. In addition, prison overcrowding (120.7 prisoners per 100 places, in 2002) has been decreasing since then; the occupancy rate in 2009 was 93.1% [16].

In accordance with the Portuguese law, offenders found not guilty by reason of insanity (non-imputable) and considered at risk of re-offending are sentenced to a security measure in which they are admitted to a security psychiatric ward, either in prison or in the public health system. They are included in the total prison population, although their number is usually small (157 individuals in 2009) [16].

In the year 2009, the prison population was 11,099 individuals, and sentenced prisoners accounted for 80.7%. The average age of prisoners was 37.8 years, which represents an ageing of four years since 2000. Young prisoners (16–20 years-old) and prisoners over 60 years-old made up 3% and 3.4% of the total population, respectively. Interestingly, the proportion of women detained has been decreasing since the year 2000, when it stood at 9.2%. It now stands at 5.4%. This trend may be due to a decrease in the number of prison sentences related to drug trafficking and an increase in the number of prison sentences related to crimes against persons.

On the other hand, the proportion of immigrants or foreigners in the prison population has been increasing, reaching one-fifth of the total in 2009 [16].

Nowadays, most prison sentences are related to crimes against property (30.5%) followed by crimes against persons (29.4%) and crimes linked to drug trafficking (22.7%). Almost half of the prisoners (49.4%) were sentenced from three to nine years of imprisonment. Prison sentences of fewer than three and more than nine years accounted for 24.7% and 23% of prisoners, respectively [16].

Healthcare in the prison system is a direct responsibility of the General-Directorate of Prison Services. In-patient care is delivered by one major prison-hospital located near Lisbon and small-size wards located in larger prisons around the national territory. Whenever the level of healthcare needed exceeds the specialization of the prison health services (e.g. intensive care, neurosurgery), prisoners are admitted to National Health Service facilities. In general, however, healthcare is delivered in each prison facility (e.g. general practice, psychiatry, nursing, dentistry), and there is an administrative policy to contract healthcare services and staff from private companies. Mental health care is mainly delivered on an “out-patient” basis at the prison facilities, and in-patient admissions are effected in two psychiatric wards, one at S. João de Deus Prison-Hospital (HPSJD), located near Lisbon, and the other at Santa Cruz do Bispo Prison, in Oporto. These facilities hold 28 and 12 beds, respectively.

An existing analysis of various surveys shows that prisoners at prison entry display high levels of morbidity, particularly communicable diseases (HIV, viral hepatitis and tuberculosis), and addictions to drugs. Addictions, suicide and mental health disorders are the major health problems, but, aside from data on drug abuse, the mental health status of Portuguese prisoners remains still largely unknown [20,21].

A recent study, including 10,182 prisoners, concluded that 40% were drug abusers and of these, half take more than one drug. *Cannabis* was the most frequent substance of abuse (52.5%), 31.8% of prisoners used cocaine intravenously, and 30.5% abused heroin [20].

For drug addiction treatment, there are seven free drug wings located in several prisons across the territory. In these special units the treatment programme aims at abstinence and lasts eighteen months on average. The programme incorporates educational, occupational and therapeutic activities. Drug addicted prisoners can also be treated at the public facilities belonging to the Drug Addiction Institute (IDT), such as therapeutic communities or outpatient clinics [21].

Over the last fourteen years suicide death rates in Portuguese prisons have fluctuated between seven and 23 deaths per year, with an average of 14.7 a year, and 11 per 10,000 prisoners. The mean suicides/overall mortality ratio is 0.185 (18.5%). Notwithstanding, the overall mortality rate has been decreasing steadily [21]. According to European statistics, in the period of 2002–2006, France ranked first, with a suicide death rate of 20 per 10,000 prisoners, and Portugal was fourth (12/10,000 prisoners). Between the two were Denmark (13.5/10,000 prisoners) and Belgium (13/10,000 prisoners) [22]. However, when the suicide rate per prisoner entries per year is calculated, the ranking position changes and Portugal becomes the leader with a rate of eight suicide deaths per 10,000 prisoners admitted per year. Another relevant calculation takes into account the suicide rate of the general population in each country and measures the excess suicide deaths in prison compared to the national rate. Again, Portugal shows one of the highest excess of suicide deaths in prison (prison/non-prison ratio of suicide rates: 8), only outranked by Italy (prison/non-prison ratio of suicide rates: 9) [22].

Given the absence of reliable forensic mental health needs assessment tools validated for the Portuguese language, the validation of the CANFOR-Research Version is a relevant step towards the improvement of care for MDOs in our country.

## Methods

### Translation and back-translation procedures

The validation procedure started with the complete translation of the English research version of CANFOR into the Portuguese language. A focus group from the psychiatric ward staff of a prison-hospital (HPSJD) was organized in order to adapt the translated version to the contextual and cultural setting of Portugal. This focus group comprised five psychiatrists, two nurses, one psychologist, and one social worker. A brief pilot-study with five opportunistic interviews to prisoners admitted at psychiatric ward was then performed to check comprehension of the questions. Finally, the corrected version was back-translated into English and checked for consistency by the first author of the original version.

### Participants

All individuals receiving care in four different forensic psychiatric facilities (forensic psychiatric services users – FPSUs) over a one year period (May 2009-May 2010) were selected. The forensic services, chosen by convenience in the Greater Lisbon region, were composed of: the psychiatric ward of a prison-hospital (28 beds with a median stay of 27 days - HPSJD), two psychiatric “out-patient” clinics (one in a male prison, Caxias Prison, and one in a female prison, Tires Prison, with 390 male and 425 female prisoners, respectively), and a civil security psychiatric ward for non-imputable offenders (31 beds in Hospital Miguel Bombarda (HMB)).

Due to circumstantial reasons, not all FPSUs participated in inter-rater and test-retest reliability procedures. As a result, there are differences between the participant numbers (n) in inter-rater, test-retest and convergent studies. All the participants signed the consent form.

### Evaluation instruments

The following instruments were included in the research protocol: a socio-demographic questionnaire designed specifically for this study; the Mini International Neuropsychiatric Interview 5.0.0 (MINI) [23], which was already in use in the prison population [24] and was included in order to validate the diagnosis using DSM-IV [25]; the Brief Psychiatric Rating Scale 4.0 (BPRS) [26], an extensively used tool to assess psychopathology; the Global Assessment of Functioning (GAF) - disability scale [27], in order to assess the level of the participants’ psychosocial functioning, in accordance with prior validating studies [14,28]^;^ and finally, the CANFOR – Portuguese research version.

### Inter-rater and test-retest reliability

Both the inter-rater and test-retest reliability studies were performed by two researchers (MT and AC). Inter-rater reliability was tested by simultaneous scoring: while one researcher conducted the interview and scored the CANFOR domains, the second one sat silently in the room and scored the CANFOR independently. The researchers’ roles alternated in consecutive interviews. A total of ninety-six FPSUs were scored using this procedure. In order to study the test-retest reliability a second CANFOR scoring was carried out after one to two weeks by the same researcher who had performed the first scoring. A total of ninety-nine FPSUs were scored using this procedure.

### Convergent validity

The convergent validity of CANFOR was studied using two instruments: the GAF - disability scale, and the BPRS. The GAF - disability scale, is rated from 10 to 100 (from lowest to highest psychosocial functioning) while the BPRS`s 22 scales of psychopathological symptoms and signs are rated between 1 (not present) and 7 (extremely severe).

### Statistical analysis

Basic descriptive statistics were used to describe the socio-demographic, clinical and forensic characteristics of the sample.

In the studies of inter-rater and test-retest reliability, the Kappa weighted coefficient was calculated for the three values of need status (no need, met need and unmet need) from the first section of CANFOR [29,30]. In order to minimise empty cells in the tables necessary for Kappa calculation, the scores “8-not applicable” and “9-unknown” were considered missing values. According to Landis and Koch [31], a Kappa coefficient of 0.4 to 0.6 indicates a moderate grade of agreement, between 0.6 and 0.8 indicates significant agreement, and between 0.8 and 1 almost perfect agreement.

The differences between CANFOR summary scores rated by FPSUs and staff were tested with Paired *t* test.

Convergent validity between CANFOR total scores and the GAF disability scale, and CANFOR domains and BPRS scales were considered using non-parametric tests (Spearman correlation and Mann–Whitney test, respectively) due to the data being skewed. All data were analysed using the Statistical Package for the Social Sciences (SPSS) for Windows, version 17.0.0 and GraphPad Software (http://graphpad.com/quickcalcs/kappa1/).

### Ethics approval

The research project was approved by the General-Directorate of Prison Services, Ministry of Justice and the Scientific Board of Faculty of Medical Sciences, New University of Lisbon.

## Results

### Participant characteristics

A total of 143 subjects, and respective case manager, were recruited. The FPSUs belonged to the following facilities: 68 (47.6%) from the prison psychiatric ward, 24 (16.8%) from the male psychiatric clinic, 22 (15.4%) from the female psychiatric clinic, and 29 (20.3%) from the civil security ward. A total of 38 (30%) subjects (36 from the prison hospital and 2 from the civil security ward) were not included mainly due to discharge, cognitive impairment or refusal (Table 1).

**Table 1:** Socio-demographic characteristics of the FPSUs

	**Socio-demographic data**	
**(n=143)**	**n**	**%**
**Gender**		
Males	108	75.5
Females	35	24.5
**Age** (mean±s.d.)		
Males	39.7 ± 12.78	
Females	35.2 ± 9.98	
**Origin**		
Prison-Hospital^1^	68	47.6
Male prison^2^	24	16.8
Female prison^3^	22	15.4
Civil security unit^4^	29	20.3
**Nationality**		
Portuguese	112	78.3
Portuguese speaking country	21	14.7
European country	7	4.9
Other	3	2.1
**Civil status**		
Single	92	64.3
Married or equivalent status	26	18.2
Divorced	19	13.3
Widowed	6	4.2
**School years**		
<4 years	20	14.0
4 years	44	30.8
6 years	23	16.1
9 years	31	21.7
12 years	19	13.3
University	6	4.2

^1^ Hospital Prisional S. João de Deus; ^2^ Estabelecimento Prisional de Caxias; ^3^ Estabelecimento Prisional de Tires; ^4^ Hospital Miguel Bombarda.

Males constituted the predominant gender of the sample. Almost half of the sample came from the psychiatric ward of the prison hospital and the majority were Portuguese citizens, were single and had attended a maximum of six years of education.

Around eighty per cent of the sample were convicted prisoners or on remand and twenty per cent were NGRI individuals detained in a civil security ward by court decision. The majority of the FPSUs (59.4%) were accused of an “offence against persons”, which includes all crimes involving violence against persons. The category “offences against property” includes crimes against material possessions, not involving violence. Almost half of the sample (49.7%) had previously been convicted and one third of the participants had received between two and five convictions before the actual sentence (Table 2).

**Table 2:** Forensic and clinical data of the FPSUs

	**Forensic and clinical data**	
**(n=143)**	**n**	**%**
**Judicial status**		
Prisoners		
Convicted	77	53.8
On Remand	37	25.9
NGRI*, security measure	29	20.3
**Actual offence**		
Offences against persons	85	59.4
Offences against property	32	22.4
Other	26	18.2
**Prior judicial history**		
No convictions	71	49.7
Suspended sentence	18	12.6
Sentenced to prison	50	35.0
Security measure	3	2.1
Unknown	1	0.7
**Number of prior convictions**		
One	8	5.6
2 to 5	48	33.6
more than 5	15	10.5
Referral reasons for prisoners** (n=114)		
Psychopathology	87	76.3
Danger to self	47	41.2
Danger to others	26	22.8
Drug withdrawal	18	15.8
Other	17	14.9
**MINI diagnostic, current** (n=114)		
Major depression	64	56.1
Major depression with melancholic features	23	20.2
Dysthymia	9	7.8
Suicide risk	50	43.9
Mania, Hypomania	11	9.6
Panic disorder	10	8.8
Agoraphobia	4	3.5
Social phobia	2	1.8
Obsessive-compulsive disorder	5	4.4
Posttraumatic stress disorder	23	20.2
Alcohol dependence/abuse	10	8.8
Non-alcohol substance dependence/abuse	54	47.4
Psychotic disorders	16	14.0
Anorexia nervosa	2	1.8
Bulimia nervosa	16	14.0
Generalized anxiety disorder	52	45.6
Antisocial personality disorder, lifetime	49	43.0

^1^ Not guilty by reason of insanity. ** One or more referral reasons.

The most common reason for referral to the psychiatric service was assessment of “psychopathological condition” (n=87; 76.3%), and the second most frequent reason was “danger to self” (n=47; 41.2%). However, if both “danger to self” and “danger to others” are considered, 64% (n=73) of FPSUs presented some form of behavioural risk (Table 2).

With regard to the current diagnosis, the most frequent MINI diagnosis was “major depression - current episode” (56.1%). About one third of these participants were found to show depression with melancholic features, and almost half were found to present positive suicide risk. The second most common diagnosis was “cannabis abuse”.

The axis II diagnosis found that 43% of the FPSUs suffered from anti-social personality disorder.

The CANFOR summary scores, the BPRS and GAF - disability scale total scores are described in Table 3. The analysis performed (paired *t* test and Spearman correlation) of CANFOR summary scores shows that the total of unmet needs obtained from FPSUs and staff ratings are significantly different, although they have a positive significant correlation. By contrast, the average of met needs display an inverse picture: the total scores between FPSUs and staff ratings were almost identical, but they did not correlate with each other. As could be anticipated, the averages of total needs, rated by FPSUs and staff, are significantly different and not correlated.

**Table 3:** Instruments` total scores rated by FPSUs and staff

	**Instruments` total scores**		
	**mean ± s.d.**	**Paired *t *test**	**Spearman´s rho**
		**p value**	**(p value)**
**CANFOR**^1^ (n=143)			
Total needs rated by FPSUs	7.8 ± 3.05	<0.001	0.122
Total needs rated by staff	6.1 ± 2.90		(0.145)
Total met needs rated by FPSUs	2.5 ± 1.85	0.926	-0.030
Total met needs rated by staff	2.5 ± 2.08		(0.722)
Total unmet needs rated by FPSUs	5.2 ± 2.65	<0.001	0.185
Total unmet needs rated by staff	3.6 ± 2.03		(0.027)
**GAF**^2^ (n=143)	46.8 ± 20.07		
**BPRS**^3^ (n=114)	38.1 ± 6.85		

^1^ – score range 0–25. ^2^ – score range 1–100 (better). ^3^ – score range 24–168 (worst).

### Inter-rater reliability

The procedures regarding inter-rater reliability were completed in 96 FPSUs. The inter-rater reliability study showed Kappa weighted coefficients of over 0.8 (almost perfect agreement) in most CANFOR domains. The domains of “Food”, “Alcohol” and “Sexual Offences” showed the lowest agreement between raters – between 0.5 and 0.8 (Table 4).

**Table 4:** I**nter-rater and Test-retest reliability data**

	**Inter-rater reliability**		**Test-retest reliability**	
	**n=96**		**n=99**	
	**No need, met need, unmet need scores**		**No need, met need, unmet need scores**	
**CANFOR domains**	% agreement	Kappa weighted coefficient	% agreement	Kappa weighted coefficient
(n = 99)				
Accommodation	100	1.000	100	*
Food	75.00	0.600	75.00	na
Living environment	98.67	0.917	86.44	0.276
Self-care	98.67	0.941	88.14	0.477
Daytime activities	81.33	0.807	55.93	0.408
Physical health	90.67	0.873	77.97	0.661
Psychotic symptoms	90.14	0.875	71.43	0.556
Information	97.33	0.974	59.32	0.369
Psychological distress	90.67	0.896	55.93	0.491
Safety to self	93.24	0.929	72.88	0.589
Safety to others	98.61	0.969	86.21	0.374
Alcohol	98.44	0.796	100	*
Drugs	97.14	0.954	92.59	0.721
Company	90.54	0.864	58.62	0.336
Intimate relationships	93.33	0.845	72.88	0.225
Sexual expression	97.26	0.940	80.70	0.608
Child care	90.91	0.862	56.00	0.231
Basic education	96.00	0.911	84.75	0.669
Telephone	95.95	0.919	79.31	0.289
Transport	94.44	0.842	100	*
Money	95.65	0.863	87.04	0.465
Benefits	95.77	0.952	83.33	0.746
Treatment	98.63	0.977	77.19	0.496
Sexual offences	80.00	0.583	33.33	0.182
Arson	100	1.000	33.33	0.182
Kappa weighted coefficient mean		0.884		0.445

* Kappa coefficient not calculated because the values of the variable are constant. na – Non applicable due to empty cells in the crosstable.

### Test-retest reliability

Test-retest reliability procedures were completed in 99 FPSUs. The average Kappa weighted coefficient was 0.445 (moderate level of agreement). Eight CANFOR domains (32%) had Kappa weighted coefficients that fell below 0.4 (fair grade of agreement): “Living environment”, “Information”, “Safety to Others”, “Company”, “Intimate Relationships”, “Child Care”, “Telephone”, and “Arson”. The Kappa weighted coefficients could not be calculated in “Accommodation”, “Alcohol” or “Transport” domains because the need status value was constant (100% agreement). In the “Food” domain the Kappa weighted coefficient was not applicable due to several empty cells in the agreement table (Table 4).

### Convergent validity

The correlation between CANFOR summary scores and GAF - disability scale were significantly negative (as CANFOR summary scores increased, the GAF score decreased) in all CANFOR scores, except for the “met needs” and “satisfaction” rated by FPSUs. Higher correlations were found in “unmet needs”, “total needs” and “formal help needed” in both users and staff ratings (Table 5).

**Table 5:** Correlations between CANFOR summary scores and GAF and BPRS

	**FPSUs rating**	**Staff rating**	**FPSUs rating**	**Staff rating**
	***vs *GAF**	***vs *GAF**	***vs *BPRS**	***vs *BPRS**
	**(n = 143)**	**(n = 143)**	**(n = 114)**	**(n = 114)**
**CANFOR summary scores**	Spearman´s rho		Spearman´s rho	
	(p value)		(p value)	
Met needs	0.009	-0.283	-0.033	0.030
	(ns)	(0.001)	(ns)	(ns)
Unmet needs	-0.385	-0.323	0.280	0.232
	(<0.001)	(<0.001)	(0.003)	(0.013)
Total needs	-0.334	-0.415	0.261	0.181
	(<0.001)	(<0.001)	(0.005)	(ns)
Informal help given	-0.177	-0.266	0.109	0.009
	(0.035)	(<0.001)	(ns)	(ns)
Formal help given	-0.169	-0.394	0.078	0.204
	(0.043)	(<0.001)	(ns)	(0.030)
Formal help needed	-0.333	-0.417	0.169	0.200
	(<0.001)	(<0.001)	(ns)	(0.033)
Satisfaction	-0.026		0.058	
	(ns)		(ns)	

ns- Not statistically significant (p>0.05).

The correlation between CANFOR summary scores and BPRS total score revealed a significant positive correlation in “unmet needs” and “total needs” scores as rated by FPSUs, and a significant positive correlation in “unmet needs”, “total needs”, “formal help given” and “formal help needed” scores as rated by the staff. The users’ satisfaction ratings showed no correlation with GAF or BPRS scores (Table 5).

Subsequently, tests were run on the presence of differences in the BPRS mean scores between the FPSUs with unmet need scores, as rated by themselves and staff, and the FPSUs without unmet need scores. The FPSUs with unmet needs in domains such as “Daytime Activities”, “Psychotic Symptoms” and “Information about Condition and Treatment” – as rated by themselves – showed significant increased means in the BPRS scales of “Hallucinations”, “Unusual Thought Content”, “Bizarre Behaviour” and “Distractibility”. Those with unmet needs in “Physical health”, “Psychological Distress”, “Safety to Self”, and “Drugs” domains showed significant increased means in the BPRS scales of “Somatic Concern”, “Anxiety”, “Depression”, “Guilt”, and “Suicide”, compared to FPSUs without unmet need scores. Another interesting finding was the significant increased means in “Hostility”, “Tension”, “Excitement”, “Uncooperativeness”, and “Motor hyperactivity” BPRS´s scales in FPSUs with unmet needs in “Safety to Others” and “Arson” domains, compared to FPSUs without unmet needs. There was considerable similarity in the significant differences between means in the BPRS scales, according to FPSUs needs status, in both user and staff ratings. In order to minimise the problems associated with multiple testing, only the significant differences between means that attained a p value less than 0.01 are displayed in Tables 6 and 7. The unmet needs in domains related to personal functioning and sexual abusive behaviour - “Looking after the living environment”, “Self-care”, “Alcohol”, “Basic education”, “Transport”, “Money”, and “Sexual Offences” - showed no differences between the means in the BPRS scale scores. Further details are available from the authors.

**Table 6:** Significant differences in BPRS scores in FPSUs with unmet need versus without unmet need score in CANFOR domains

		**BPRS**		
**CANFOR**	**User´s ratings**	**Scales**	**Median (Mean)**^**a**^	**Mann–Whitney *U *test**
Domains*	**0= other score (n)**	**(range: 1–7)**		
**(n=114)**	**1=unmet need (n)**			**p value**
**1. Accommodation**	0 (110)	**Motor hyperactivity**	1.00	0.001
	1 (4)		2.00	
**2. Food**	0 (112)	**Excitement**	1.00	0.001
	1 (2)		4.50	
	0 (112)	**Motor hyperactivity**	1.00	<0.001
	1(2)		3.50	
**5. Daytime activities**	0 (57)	**Hallucinations**	1.00 (1.25)	<0.001
	1 (57)		1.00 (1.93)	
	0 (57)	**Unusual thought content**	1.00 (1.12)	<0.001
	1 (57)		1.00 (1.79)	
**6. Physical health**	0 (92)	**Somatic concern**	1.00	<0.001
	1 (22)		3.00	
**7. Psychotic symptoms**	0 (101)	**Hallucinations**	1.00	<0.001
	1 (13)		3.00	
	0 (101)	**Unusual thought content**	1.00	<0.001
	1 (13)		3.00	
	0 (101)	**Bizarre behaviour**	1.00 (1.18)	0.001
	1 (13)		1.00 (2.00)	
	0 (101)	**Uncooperativeness**	1.00 (1.01)	<0.001
	1 (13)		1.00 (1.23)	
	0 (101)	**Distractibility**	1.00 (1.01)	0.002
	1 (13)		1.00 (1.23)	
**8. Information about condition and treatment**	0 (63)	**Hallucinations**	1.00 (1.32)	<0.001
	1 (51)		1.00 (1.92)	
	0 (63)	**Unusual thought content**	1.00 (1.14)	<0.001
	1 (51)		1.00 (1.84)	
**9. Psychological distress**	0 (42)	**Anxiety**	3.00 (2.60)	<0.001
	1 (72)		3.00 (3.35)	
	0 (42)	**Depression**	2.00	<0.001
	1 (72)		4.00	
	0 (42)	**Suicidality**	1.00	<0.001
	1 (72)		2.00	
**10. Safety to self**	0 (70)	**Depression**	3.00	0.007
	1 (44)		4.00	
	0 (70)	**Suicidality**	1.00	<0.001
	1 (44)		4.00	
**11. Safety to others**	0 (99)	**Hostility**	1.00	<0.001
	1 (15)		4.00	
	0 (99)	**Tension**	1.00	<0.001
	1 (15)		3.00	
**13. Drugs**	0 (88)	**Suicidality**	1.00	0.004
	1 (26)		3.50	
	0 (88)	**Guilt**	2.00	0.003
	1 (26)		3.00	
**14. Company**	0 (66)	**Somatic concern**	1.00	0.005
	1 (48)		2.00	
	0 (66)	**Motor retardation**	1.00 (1.11)	0.001
	1 (48)		1.00 (1.42)	
	0 (66)	**Excitement**	1.00 (1.48)	0.003
	1 (48)		1.00 (1.10)	
**15. Intimate relationships**	0 (69)	**Guilt**	2.00	0.005
	1 (45)		3.00	
**16. Sexual expression**	0 (39)	**Guilt**	2.00	0.007
	1 (75)		3.00	
	0 (39)	**Hostility**	1.00	0.009
	1 (75)		2.00	
	0 (39)	**Suspiciousness**	2.00 (1.77)	0.009
	1 (75)		2.00 (2.31)	
**19. Telephone**	0 (103)	**Disorientation**	1.00 (1.00)	0.002
	1 (11)		1.00 (1.09)	
**22. Social benefits**	0 (31)	**Motor retardation**	1.00 (1.00)	0.002
	1 (83)		1.00 (1.33)	
**23. Treatment**	0 (105)	**Grandiosity**	1.00 (1.14)	<0.001
	1 (9)		1.00 (2.33)	
	0 (105)	**Bizarre behaviour**	1.00 (1.22)	0.009
	1 (9)		1.00 (1.89)	
**25. Arson**	0 (110)	**Excitement**	1.00	0.010
	1 (4)		2.00	
	0 (110)	**Motor hyperactivity**	1.00	0.001
	1 (4)		1.50	

* Unmet needs scores in **3.Looking after the living environment, 4.Self-care, 12.Alcohol, 14. Company, 17.Child Care, 18.Basic education, 20.Transport, 21.Money** and **24.Sexual Offences** domains without high significant associations with BPRS scales (>0.01). ^a^ Calculation of the mean whenever median is the same in (0) and (1) scores.

**Table 7:** Significant differences in BPRS scores in FPSUs with unmet need versus without unmet need score, rated by staff, in CANFOR domains

		**BPRS**		
**CANFOR**	**Staff´s ratings**	**Scales**	**Median (Mean)**^**a**^	**Mann–Whitney *U *test**
Domains*	**0= other score (n)**	**(range: 1–7)**		
**(n=114)**	**1=unmet need (n)**			**p value**
**1. Accommodation**	0 (112)	**Elated mood**	1.00	0.006
	1 (2)		3.50	
**6. Physical health**	0 (104)	**Somatic concern**	1.00	0.009
	1 (10)		3.00	
**7. Psychotic symptoms**	0 (109)	**Hallucinations**	1.00	<0.001
	1 (5)		3.00	
	0 (109)	**Unusual thought content**	1.00	<0.001
	1 (5)		3.00	
	0 (109)	**Bizarre behaviour**	1.00 (1.18)	<0.001
	1 (5)		1.00 (2.00)	
	0 (109)	**Blunted affect**	1.00 (1.07)	<0.001
	1 (5)		1.00 (1.31)	
	0 (109)	**Emotional withdrawal**	1.00 (1.08)	0.004
	1 (5)		1.00 (1.15)	
	0 (109)	**Uncooperativeness**	1.00 (1.01)	<0.001
	1 (5)		1.00 (1.23)	
	0 (109)	**Distractibility**	1.00 (1.01)	<0.001
	1 (5)		1.00 (1.23)	
**8. Information about condition and treatment**	0 (106)	**Hallucinations**	1.00 (1.32)	<0.001
	1 (8)		1.00 (1.92)	
	0 (106)	**Unusual thought content**	1.00 (1.14)	<0.001
	1 (8)		1.00 (1.84)	
	0 (106)	**Bizarre behaviour**	1.00 (1.11)	<0.001
	1 (8)		1.00 (1.47)	
	0 (106)	**Blunted affect**	1.00 (1.08)	0.001
	1 (8)		1.00 (1.12)	
	0 (106)	**Uncooperativeness**	1.00 (1.03)	0.001
	1 (8)		1.00 (1.04)	
**9. Psychological distress**	0 (59)	**Depression**	2.00	0.002
	1 (55)		4.00	
	0 (59)	**Suicidality**	1.00	0.007
	1 (55)		2.00	
**10. Safety to self**	0 (87)	**Depression**	3.00	0.003
	1 (27)		4.00	
	0 (87)	**Suicidality**	1.00	<0.001
	1 (27)		4.00	
**11. Safety to others**	0 (91)	**Hostility**	1.00	<0.001
	1 (23)		4.00	
	0 (91)	**Tension**	1.00	0.001
	1 (23)		3.00	
	0 (91)	**Excitement**	1.00 (1.30)	0.007
	1 (23)		1.00 (1.47)	
**13. Drugs**	0 (84)	**Suicidality**	1.00	0.001
	1 (30)		3.50	
	0 (84)	**Guilt**	2.00	0.007
	1 (30)		3.00	
**17. Child care**	0 (80)	**Anxiety**	3.00 (3.08)	0.003
	1 (34)		3.00 (3.05)	
**22. Social benefits**	0 (106)	**Disorientation**	1.00 (1.00)	<0.001
	1 (8)		1.00 (1.01)	

* Unmet needs scores in **3.Looking after the living environment, 4.Self-care, 5.Daytime activities, 12.Alcohol, 14.Company, 15.Intimate relationships, 16.Sexual expression, 17.Child Care, 18.Basic education, 20.Transport, 21.Money, 22.Treatment, 24.Sexual Offences** and **25.Arson** domains without high significant associations with BPRS scales (>0.01). Associations between unmet needs scores in **2.Food** and **19.Telephone** domains and BPRS scales not calculated due to variable constant. ^a^ Calculation of the mean whenever median is the same in (0) and (1) scores.

## Discussion

### Inter-rater reliability study

The CANFOR Portuguese version, which was based on the research version, showed an inter-rater reliability that was very similar to the original [14] and Spanish versions [23]. Only in three domains (12%) was the level of inter-rater agreement below 0.8 (almost perfect agreement). This high inter-rater agreement level could be due to several reasons: the ease with which the respondents understood the questions, the ease with which the raters understood the need status categories, and previous training on the instrument. In addition, the two researchers who carried out the interviews in our study had participated in the preparation of the Portuguese version of CANFOR from the beginning and shared the training on the instrument.

### Test-retest reliability study

The test-retest reliability study revealed a moderate level of agreement (Kappa coefficient average between 0.4-0.6). The inferior test-retest reliability, compared to inter-rater reliability, could be related to rapid changes in FPSUs’ opinions/perceptions caused by stress in prison or security facilities. In addition, it is understandable that mood state, anxiety and other psychopathology from FPSUs could modify their opinions/perceptions of individual needs. This difference in agreement level between inter-rater and test-retest reliability studies was also found in the original validation study [14].

### Convergent validity

In previous validation studies [14,23], due to the lack of other instruments suited to assessing individual need levels in a forensic setting, convergent validation was conducted with the GAF and other instruments that evaluate several aspects of disability. In the present study it was decided to use GAF - disability scale and the BPRS. The results demonstrated that individuals with more needs, especially unmet needs, rated higher scores of disability. This finding is similar to the results of other validation studies, and it shows an inverse relationship between the level of individual needs and the level of psychosocial functioning.

With regard to convergence between CANFOR and BPRS, there was a significant positive correlation of “unmet needs” and “total needs” scores, as rated by FPSUs and staff, and BPRS total scores. These results suggest a direct relationship between the level of individual unmet needs and the global psychopathology. The analysis of convergence between CANFOR domains’ unmet needs and BPRS scales scores revealed interesting results. In seventeen and ten CANFOR domains, as rated by self and staff respectively, a significant higher mean score in several psychopathological scales in FPSUs with unmet needs was found (Tables 6 and 7). This was particularly evident in CANFOR domains addressing health status and behavioural risks, namely “Physical Health”, “Psychotic Symptoms”, “Information about Condition and Treatment”, “Psychological Distress”, “Safety to Self”, “Safety to Others”, and “Drugs”. The congruence between specific domains’ unmet needs and severity of psychopathology emphasizes the construct validity of CANFOR as a tool for individual care needs assessment. Moreover, these results underlined the importance of psychiatric evaluation and intervention in the general care planning directed to MDOs while detained in prison or security facilities.

### Strengths and limitations

This study, which sought to assess the validity and reliability of the CANFOR Portuguese version - research version, was embedded in a broader research project that aims to evaluate the care needs in mentally ill offenders while in prison. This focus resulted in some advantages and shortcomings. Due to reasons of convenience, we chose prisons and security facilities in the Greater Lisbon area. Considering the size of the target-population, we decided to enrol all the FPSUs in a twelve month period instead of randomising them. This procedure made it possible to perform a structured assessment of both genders from different forensic psychiatric facilities (a prison psychiatric ward, prison psychiatric clinics and a civil security ward) in a substantial number of FPSUs. As a result the sample was heterogeneous and close to the MDO reality. In order to evaluate associations between unmet needs of care (CANFOR) and psychopathology we included the BPRS in the research protocol. However, the workload prevented the CANFOR staff reliability study. Nevertheless, we think that this limitation should be balanced taking into consideration that CANFOR reliability is not affected by the respondent status – service user or professional because in the previous studies [14,23] there have been no relevant differences of reliability data involving FPSUs or staff. To our knowledge, this is the first convergence study using these instruments in a forensic sample and the first standardized assessment of care needs in Portuguese detainees.

## Conclusions

The CANFOR Portuguese version, rated by FPSUs, revealed similar psychometric properties to the original version [14]. The study of convergent validity of CANFOR with the GAF - disability scale and BPRS shows statistically significant results, which confirm that this tool is based on sound constructs and is appropriate for individual care needs assessment in this population.

The number of unmet needs differs significantly between FPSUs and staff. Although this fact is not related to the tool’s reliability, it demonstrates the usefulness of CANFOR to evaluate care needs in the service user’s perspective. The CANFOR Portuguese version was straightforward to administer and proved to be a reliable tool to identify clinical and social problems in FPSUs. In brief, CANFOR has the potential to be used by a range of different professionals and was considered to be a valuable tool to guide and to evaluate the health and social interventions in a mental health forensic setting.

## Competing interests

The authors declare that they have no competing interests.

## Authors’ contributions

MT conceived the study, participated in the study design, data collection, statistical analysis and the draft manuscript. ST reviewed the manuscript and approved the CANFOR Portuguese version in order to be published. AC participated in data collection, statistical analysis and the draft manuscript. PA contributed to statistical analysis and the draft manuscript. JMCA participated in the study design and the draft manuscript. MX participated and coordinated the study design, data collection, statistical analysis and the draft manuscript. All authors read and approved the final manuscript.

## Pre-publication history

The pre-publication history for this paper can be accessed here:

http://www.biomedcentral.com/1471-244X/13/157/prepub
